# Contribution of Pore-Connectivity to Permeation Performance of Silicalite-1 Membrane; Part I, Pore Volume and Effective Pore Size

**DOI:** 10.3390/membranes11060382

**Published:** 2021-05-24

**Authors:** Motomu Sakai, Yukichi Sasaki, Takuya Kaneko, Masahiko Matsukata

**Affiliations:** 1 Research Organization for Nano & Life Innovation, Waseda University, 513 Wasedatsurumaki-cho, Shinjuku-ku, Tokyo 162-0041, Japan; mmatsu@waseda.jp; 2 Nanostructures Research Laboratory, Japan Fine Ceramics Center, 2-4-1 Atsuta-ku, Nagoya-shi, Aichi 456-8587, Japan; sasaki@jfcc.or.jp; 3 Department of Applied Chemistry, Waseda University, 513 Wasedatsurumaki-cho, Shinjuku-ku, Tokyo 162-0041, Japan; t.kaneko.waseda@gmail.com; 4 Advanced Research Institute for Science and Engineering, Waseda University, 513 Wasedatsurumaki-cho, Shinjuku-ku, Tokyo 162-0041, Japan

**Keywords:** silicalite-1, membrane, separation, diffusion, adsorption, micropore, connectivity

## Abstract

The micropore volumes and effective pore sizes of two types of silicalite-1 membranes were compared with those of a typical silicalite-1 powder. The silicalite-1 membrane with fewer grain boundaries in the membrane layer showed similar micropore volume and effective pores size to those of the silicalite-1 powder. In contrast, when the silicalite-1 membrane contained many grain boundaries, relatively small micropore volume and effective pore size were observed, suggesting that narrowing and obstruction of the micropore would occur along grain boundaries due to the disconnection of the zeolite pore. The silicalite-1 membrane with fewer grain boundaries exhibited relatively high permeation properties for C_6_-C_8_ hydrocarbons. There was an over 50-fold difference in benzene permeance between these two types of membranes. We concluded that it is important to reduce grain boundaries and improve pore-connectivity to develop an effective preparation method for obtaining a highly permeable membrane.

## 1. Introduction

Membrane separation draws attention as a novel, energy-saving technology for separation and purification. A large quantity of energy was consumed for hydrocarbon separation in the chemical industry. Membrane separation is expected to have a huge impact on energy efficiency in this field [1]. Inorganic membranes have an advantage for hydrocarbon separation because of their high chemical and thermal resistance compared to organic membrane materials.

Zeolites are widely used as solid acid catalysts in the petroleum industry through the utilization of their shape-selectivity and acid property. Uniform micropores of zeolite—defined by their crystal structures—contribute to the appearance of their unique molecular sieving properties, which is why zeolites have been expected to function as a molecular sieving membrane material for hydrocarbon mixtures. MFI-type zeolite has two channels, one of them is a straight pore along the *b*-axis and another is a sinusoidal pore along the *a*-axis [2,3]. MFI-type zeolite membranes were studied previously for the separation of C_4_-C_8_ hydrocarbons, such as butane isomers, hexane isomers and xylene isomers [4,5,6,7,8,9,10,11,12,13]. In addition, alcohol recovery from an aqueous solution was also carried out by using an MFI-type zeolite membrane [14,15]. Although some membranes have exhibited excellent separation performance, the flux is still too low to make them practical. Therefore, the improvement of flux is one of the most important issues in membrane development.

Controlling crystal orientation and reducing membrane thickness have been attempted to improve the permeability of the MFI-type zeolite membrane. To prepare highly permeable membranes, signature methods for orientational seeding and morphology control were developed. Lai et al. reported a preparation method of oriented silicalite-1 membranes [16,17,18]. In their study, the *b*-oriented silicalite-1 membrane, in which through-pores are formed in the direction of molecular permeation, showed high *p*-xylene permeability for xylene isomer separation. Hedlund et al. prepared an ultra-thin MFI-type zeolite membrane by using a masking technique [19,20]. Pores of porous support were plugged with polymethyl methacrylate (PMMA) prior to crystallization to avoid the formation of amorphous and crystalline materials inside the support. After the crystallization, PMMA was removed by calcination. Their ultra-thin membrane exhibited high permeabilities for C_4_-C_8_ hydrocarbon separation. Recently, a 2-dimensional nano-sheet MFI-type zeolite membrane was developed by Tsapatsis and his co-workers [21]. The nano-sheet had an *a*-*c* plane, and the thickness along the *b*-axis was below 100 nm. Their *b*-oriented nano-sheet membrane had a superior *p*-xylene permselectivity. Ueno et al. reported the *b*-oriented tubular silicalite-1 membrane by a gel-free, steam-assisted conversion method. In this method, the membrane layers that retain the orientation of seed crystals can be obtained [22].

The grain boundary is also an important factor for the zeolite membrane. There is some literature that reported the effect of the grain boundary on the permeation and separation performances of the zeolite membranes. The transport barrier at the intercrystalline regions in the A-type zeolite membrane has been proposed by Kärger et al. for the first time in experimental research [23]. Takaba et al. also reported the effect of the sub-nanometer grain boundary in all-silica chabazite (CHA) zeolite membranes on their permeation properties through a calculational study [24]. In addition, the direct observation method for the grain boundary, through using fluorescene confocal optical microscopy, was reported by Tsapatsis et al. [25]. Falconer et al. reported that the grain boundary in the MFI-type zeolite membrane played an important role in hydrocarbon permeation [26]. The grain boundary in their membrane, called the “nano-valve”, opened and closed with and without the adsorption of *n*-hexane. The importance of the grain boundary for zeolite membranes has been pointed out, as mentioned above; however, the role of the grain boundary is still an open question.

**Figure 1:**
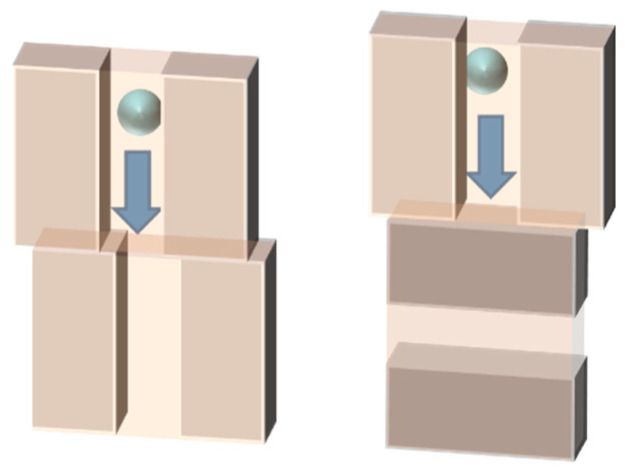
Schematic diagram of the narrowing and obstruction of micropores along the grain boundary.

Based on the results of these previous studies, we considered that the pore-connectivity in a membrane is also an important parameter for permeability. In this study, we paid attention to the grain boundary between zeolite crystals in the membrane. The micropores of zeolites would become narrower and obstructed at the grain boundaries because crystals do not necessarily form boundaries where micropores fit well with each other, as shown in Figure 1.

The disconnection of the micropore at a grain boundary would decrease the micropore volume and effective pore size. Previously, we prepared a silicalite-1 membrane that had fewer grain boundaries in the direction that disturbs molecular permeation [27]. In this study, we prepared silicalite-1 membranes with different amounts of grain boundary and investigated the pore-connectivity of these membranes using microscopic observation, nano-permporometry, physical adsorption and a single vapor permeation test. We will discuss the effect of the microstructure on the pore-connectivity and permeability in silicalite-1 membranes.

## 2. Materials and Methods

### 2.1. Procedure of Membrane Preparation

Two types of silicalite-1 membranes with different crystal stacking structures were prepared on a tubular -alumina support (i.d. = 7.0 mm, o.d. = 10 mm, length = 30 mm, average pore size = 150 nm, NORITAKE Ltd., Japan) by a secondary growth method. A dip-coating method was utilized to support the silicalite-1 seed crystals.

The preparation procedure of silicalite-1 membrane with fewer grain boundaries in the direction perpendicular to permeation was as follows: silicalite-1 powder for dip-coating slurry was prepared through the hydrothermal crystallization of the solution, with the composition of 10SiO_2_: 1.6TPAOH: 440H_2_O: 40EtOH: 0.04Na_2_O at 373 K for 24 h [28]. The solution was prepared by mixing tetraethyl orthosilicate (TEOS, 98 wt%, Merck Co., Germany), a tetrapropylammonium hydroxide solution (TPAOH, 1.0 M, Sigma-Aldrich Co., US), distilled water and sodium hydroxide (>97 wt%, Kanto Chemical Co., Japan). The powdery crystals synthesized and were dispersed in an appropriate amount of distilled water to form the slurry.

The support was dipped in the slurry for 1 min, withdrawn vertically at ca. 3 cm s^−1^ and then dried at 343 K over 40 min. This process was run twice. Both ends of tubular support were plugged with PTFE caps in order to avoid the penetration of slurry. The seeded tubular support was placed vertically in a PTFE-lined stainless autoclave with a synthesis solution. The synthesis solution with the molar composition of 10SiO_2_: 1.2TPAOH: 660H_2_O: 80EtOH was prepared by mixing TEOS, TPAOH, distilled water and ethanol (99.5 wt%, Kanto Chemical Co., Japan). This solution was aged at 333 K for 4 h while stirring prior to use. The autoclave was closed and placed in a preheated oven for hydrothermal crystallization at 373 K for 7 days. After the crystallization, the membrane was washed with boiling water and then calcined at 773 K for 8 h to remove the TPA cation occluded in the framework of the zeolite. Details of the synthesis procedure have been described elsewhere [27]. This membrane was named S-1_S_ from the initial letter of “single-layer” because single-layer crystals formed on the external surface of the support.

Another type of silicalite-1 membrane, named S-1_M_ as an acronym for “multi-layer”, was prepared as follows. S-1_M_ possessed a larger amount of grain boundaries across the permeation direction than S-1_S_. While the preparation procedure of S-1_M_ was similar to that of S-1_S_, we used different seed slurry and crystallization conditions. In this case, the silicalite-1 powder was prepared based on the methods reported in the literature [18]. A synthesis mixture with the composition of 10SiO_2_: 2TPAOH: 1000H_2_O: 40EtOH was crystallized at 403 K for 12 h, and then the obtained silicalite-1 powder was ground in an agate mortar for 24 h. The crushed powder was used to form a seed slurry. Then, the support seeded with ground silicalite-1 crystals was crystallized in the synthesis solution that had the molar composition of 10SiO_2_: 0.8TPABr: 2.6Na_2_O: 640H_2_O. This synthesis solution was prepared by mixing colloidal silica (ST-S, Nissan Chemical Ind. Ltd., Japan), tetrapropylammonium bromide (Tokyo Chemical Ind. Co. Ltd., Japan), sodium hydroxide (>97 wt%, Kanto Chemical Co. Inc., Japan) and distilled water. The hydrothermal crystallization was performed at 443 K for 24 h.

### 2.2. Nano-Permporometry

Nano-permporometry was performed to estimate the size of through-pores in the silicalite-1 membrane that was prepared [29]. The permeance of inert gas through the membrane was measured with the increasing relative pressure of condensable vapor in a stepwise manner. In this process, pores in the membrane were plugged by the adsorption and condensation of vapor in the order of pore size from small to large; thus, the permeance of inert gas decreased with the progress of measurement. In this work, helium and *n*-hexane were used for the inert gas and condensable vapor, respectively. After the thermal treatment at 573 K for 3 h to remove any adsorbed substances in the membrane, the nano-permporometry measurement was performed by the Porometer nano-6 (Microtrac-BEL Corp., Japan) at 333 K.

### 2.3. Vapor Permeation Test

The fluxes of C_6_ hydrocarbons through two types of silicalite-1 membranes in unary systems were evaluated to estimate the through-pore size. The probe molecules used were *n*-hexane, 2-methylpentane, benzene, cyclohexane and 2,2-dimethylbutane. These hydrocarbons were pumped into a preheater for vaporization and were fed to the outer surface of the tubular membrane at 10 kPa with the carrier gas helium. Because the diameter and effective length were 10 and 20 mm, the effective membrane area was 6.28 × 10^−4^ m^2^. The permeation side was swept by flowing helium. Both the feed and permeate sides were kept at atmospheric pressure. All measurements were carried out at 573 K.

The permeate flow rate was evaluated by using the gas composition at the outlet. Methane was added at the outlet as the internal standard gas. A gas chromatograph (GC-8A, Shimadzu Corp., Japan) was used to analyze the composition. Permeance, *Π*, was calculated using the following Equation (1):

*Π* = *u* · *A*^−1^ · *p*^−1^ (1)

where *u* is the permeate flow rate (mol · s^−1^), *A* is the effective membrane area (m^2^) and Δ*p* is the partial pressure difference between the feed and permeate sides (Pa).

### 2.4. Adsorption Measurement

N_2_, *n*-hexane and 2-methylpentane adsorption measurements were performed non-destructively through the use of a volumetric gas adsorption method with BELSORP-max (MicrotracBEL Corp., Japan) to determine the micropore volumes of the membranes that were prepared. A special sample holder developed in our laboratory enabled us to insert a whole zeolite membrane with support and without destruction [30]. The samples were outgassed at 623 K for 8 h under vacuum conditions prior to the adsorption test. Adsorption measurements were carried out at 77 and 298 K for N_2_ and hydrocarbons, respectively. The adsorbed amounts on membranes were calculated by dividing the adsorbed volume by the weight of the zeolite layer formed on the support.

## 3. Results

### 3.1. XRD and Microscopic Observation for Membranes

**Figure 2:**
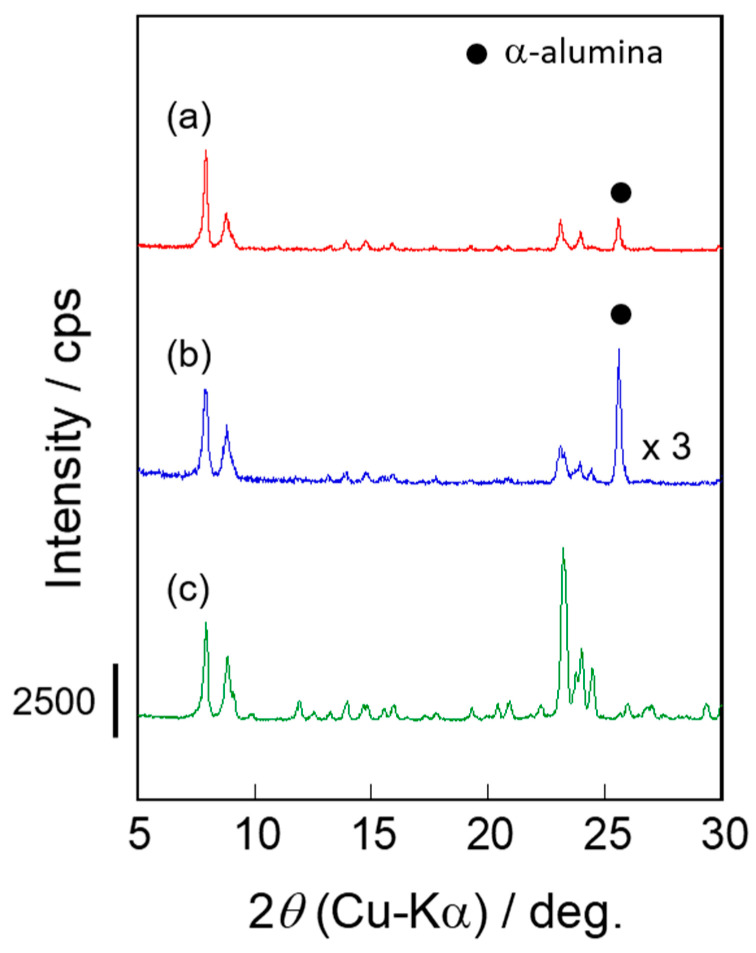
XRD patterns of silicalite-1 (**a**) S-1_S_, (**b**) S-1_M_ and (**c**) silicalite-1 powder.

We compared two types of silicalite-1 membranes prepared through the different synthesis recipes. The seeded supports and crystallized membranes were characterized by using FE-SEM and TEM images and X-ray diffraction patterns. Figure 2 draws the XRD patterns of the silicalite-1 powder and membranes.

**Figure 3:**
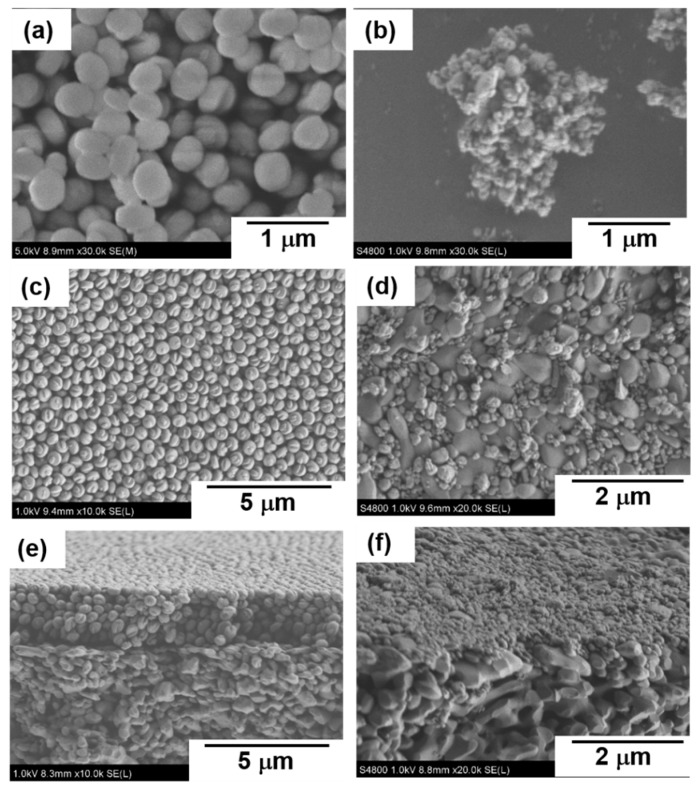
Typical FE-SEM images of seed crystals and seeded supports. (**a**,**b**) seed crystals for S-1_S_ and S-1_M_; (**c**,**d**) the surface of seeded supports for S-1_S_ and S-1_M_. (**e**,**f**) the cross-section of seeded supports for S-1_S_ and S-1_M_.

Figure 3a,b show the typical FE-SEM images of the seed crystals used in the membrane preparations. The seed crystals used for the preparation of S-1_S_ were spherical with a diameter of about 300 nm. In contrast, the seed crystals for S-1_M_ were aggregates of small crystals of ca. 100 nm. The particle size in the prepared seed slurry was also measured through the dynamic light scattering method (ELSZ-1000ZS, Otsuka Electronics, Japan). The particle sizes in the seed slurries for the preparation of S-1_S_ and S-1_M_ were 270 and 104 nm, respectively. The particle sizes measured with DLS were almost the same as the crystal size observed in the FE-SEM images in both cases of S-1_S_ and S-1_M_, suggesting that both seed crystals were well dispersed in the seed slurry. It is noted that the particle sizes in the slurries for S-1_S_ and S-1_M_ were larger and smaller than 150 nm of the average pore size of support, respectively.

Figure 3c–f show the typical FE-SEM images of the seeded supports. Spherical seed crystals uniformly covered the support after the dip-coating with the slurry for S-1_S_. In addition, seed crystals were observed only on the outer surface of the support in the cross-section image. In contrast, a small amount of crystal was observed sparsely on the surface of the support that was dip-coated with the slurry for S-1_M_ preparation.

**Figure 4:**
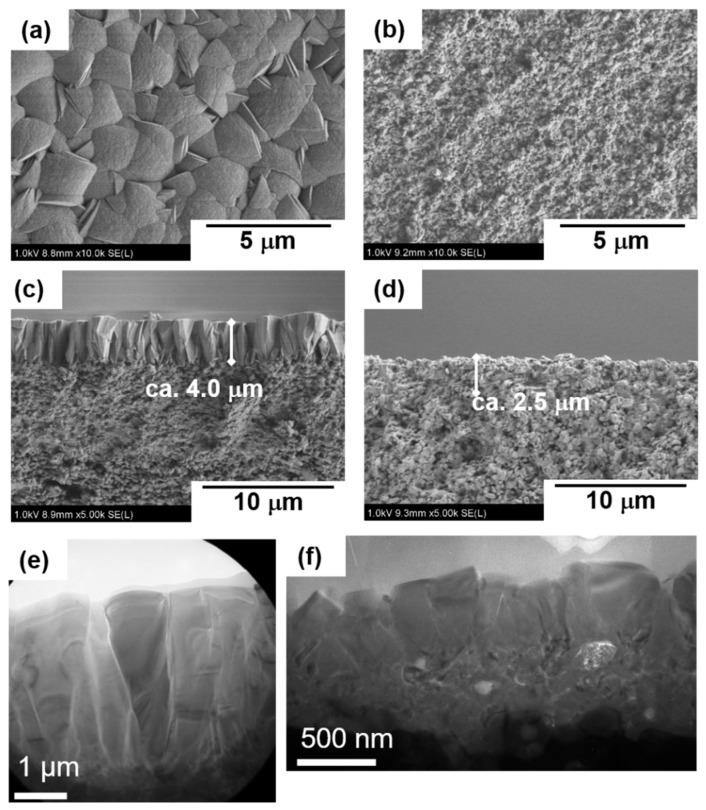
Typical FE-SEM images of silicalite-1 membranes. (**a**,**c**,**e**) S-1_S_, (**b**,**d**,**f**) S-1_M_.

Figure 4 shows the typical FE-SEM and TEM images of the two types of silicalite-1 membranes. In S-1_S_, the columnar-shaped crystals aligned in the outer layer of the membrane, as shown in Figure 4c,e. A seed crystal layer was also observed beneath the grown crystal layer. The crystal shape in the remaining seed layer was not greatly changed even after the crystallization. The grown crystals on the outer surface were arranged, and the grain boundaries were observed, along with the axis of molecular permeation. On the other hand, in S-1_M_, small crystals of ca. 500 nm, were stacked, as shown in Figure 4d,f. Consequently, many intricate grain boundaries existed in the crystal layer, in contrast to S-1_S_. The weights of the synthesized membrane layers in S-1_S_ and S-1_M_ were 68.6 and 43.7 g m^−2^, and the membrane thicknesses of the outer surface were 4.0 and 2.5 m, respectively. Therefore, we consider that most of the crystals in S-1_M_ formed inside the support.

### 3.2. Nano-Permporometry

Nano-permporometry was performed to measure the through-pore size distribution of the two types of silicalite-1 membranes. We used the Kelvin Equation (2), to calculate the pore size in the prepared membranes from the results of nano-permporometry.

*D_K_* = −4 · *νσ cosθ* · *R*^−1^*T*^−1^ · ln(*p* · *ps*^−1^)^−1^ (2)

*D_K_*, *ν*, *σ* and *θ* represent the Kelvin diameter (m), molar volume (m^3^ mol^−1^), surface tension (N m^−^^1^) and contact angle (degree), respectively. Here, *ν* and *σ* are 1.31 × 10^−4^ m^3^ mol^−1^ and 20.4 × 10^−3^ N m^−^^1^ [29,30]. Because the silicalite-1 membrane is extremely hydrophobic, we used *n*-hexane as a condensable vapor, and the contact angle *θ* was assumed as 0°. *p* · *p_S_*^−^^1^ was the relative pressure of the condensable vapor at the measuring temperature of 333 K. This equation is based on macroscopic thermodynamics and may lose its physical meaning in extremely small pores, such as zeolite pores. However, there are some previous reports about an empirical agreement of the Kelvin equation to micropores [31,32,33].

**Figure 5:**
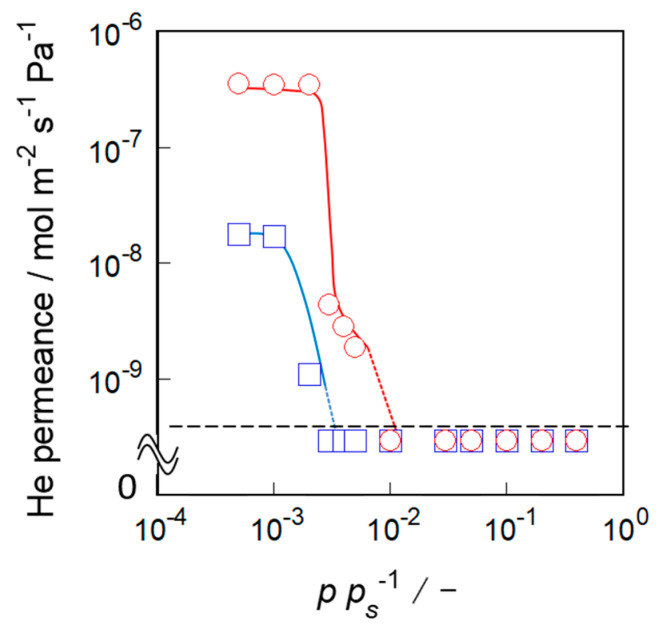
Results of the nano-permporometry for silicalite-1 membranes of S-1_S_ (○) and S-1_M_ (□). The dashed line shows the limit of quantification (5 × 10^−10^ mol m^−2^ s^−1^ Pa^−1^).

Figure 5 shows the results of the nano-permporometry for the membranes prepared. The helium permeance through S-1_S_ readily decreased at *p* · *p_S_*^−1^ = 3.0 × 10^−3^ and was below the limit of quantification (5 × 10^−10^ mol m^−2^ s^−1^ Pa^−1^) above *p* · *p_S_*^−1^ = 1.0 × 10^−2^. In contrast, the permeance through S-1_M_ steeply decreased in the earlier stage, at *p* · *p_S_*^−1^ = 2.0 × 10^−3^, and fell below the quantification limit, at *p* · *p_S_*^−1^ = 3.0 × 10^−3^. It is notable that the helium permeance through S-1_S_ and S-1_M_ at *p* · *p_S_*^−1^ = 0 were 4.48 × 10^−7^ and 8.46 × 10^−8^ mol m^−2^ s^−1^ Pa^−1^, respectively. In other words, S-1_S_ had a higher permeation performance.

### 3.3. Adsorption Measurement for Silicalite-1 Powder and Membranes

N_2_, *n*-hexane and 2-methylpentane adsorption measurements were performed for the silicalite-1 powder and the two types of membranes to compare accessible micropore volumes.

**Figure 6:**
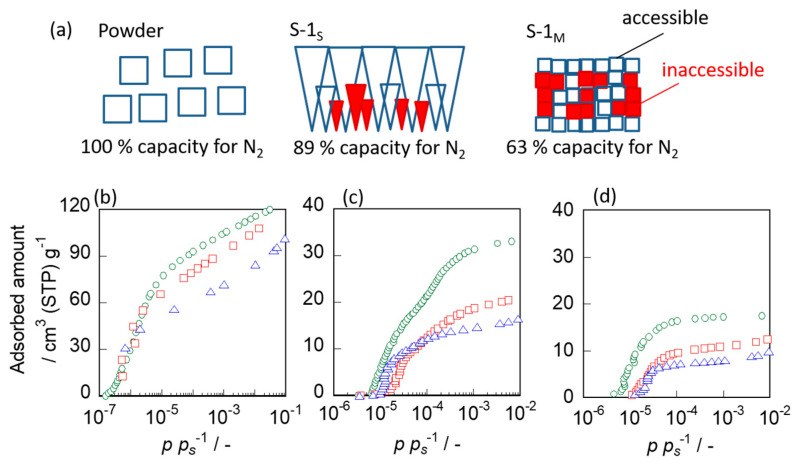
(**a**) Schematic diagram of accessible volumes in the powder and membrane. Adsorption isotherms of (**b**) N_2_, (**c**) *n*-hexane and (**d**) 2-methylpentane for ○, silicalite-1 powder; □, S-1_S_; ▵, S-1_M_.

Figure 6 shows the adsorption isotherms of N_2_, *n*-hexane and 2-methylpentane for the silicalite-1 powder and the membranes. The amount of N_2_ adsorbed at *p* · *p_S_*^−1^ = 1.0 × 10^−4^ was adopted as the saturated amount adsorbed in the zeolite pore because it represents the saturated amount of N_2_ adsorbed by the cylindrical pore with a diameter of 0.55 nm, which was almost the same as the pore diameter of the MFI-type zeolite, according to the Saito-Foley model [34,35]. Further, the amounts of *n*-hexane and 2-methylpentane adsorbed at *p* · *p_S_*^−1^ = 1.0 × 10^−2^, where the adsorption isotherms flatlined, were adopted as the saturated amount adsorbed in the zeolite pore. Table 2 compared the amounts of N_2_, *n*-hexane and 2-methylpentane adsorbed in the pore of the zeolite in the forms of powder and membranes. In addition, the relative adsorbed amount, which is the amounts adsorbed on the membranes divided by that on the powder, is also listed.

Since the amount of N_2_ adsorbed in the zeolite pore of silicalite-1 powder, S-1_S_ and S-1_M_, were 92.6, 82.1 and 58.4 cm^3^ (STP) g^−1^, respectively, the relative amounts of N_2_ adsorbed on S-1_S_ and S-1_M_ were calculated to be 0.887 and 0.631, respectively. On the other hand, the amounts of 2-methylpentane adsorbed on the powder and membranes (S-1_S_ and S-1_M_) were 19.9, 12.0 and 9.52 cm^3^ (STP) g^−1^, respectively, and the relative adsorption amounts determined from these values were 0.603 and 0.478, respectively. The amounts of N_2_, *n*-hexane and 2-methylpentane adsorbed on these membranes were all less than those on the powder. Additionally, it is also worth noting that for both types of membranes, the larger the molecule, the smaller the relative adsorbed amount tends to be.

### 3.4. Vapor Permeation Tests in Unary Systems

**Figure 7:**
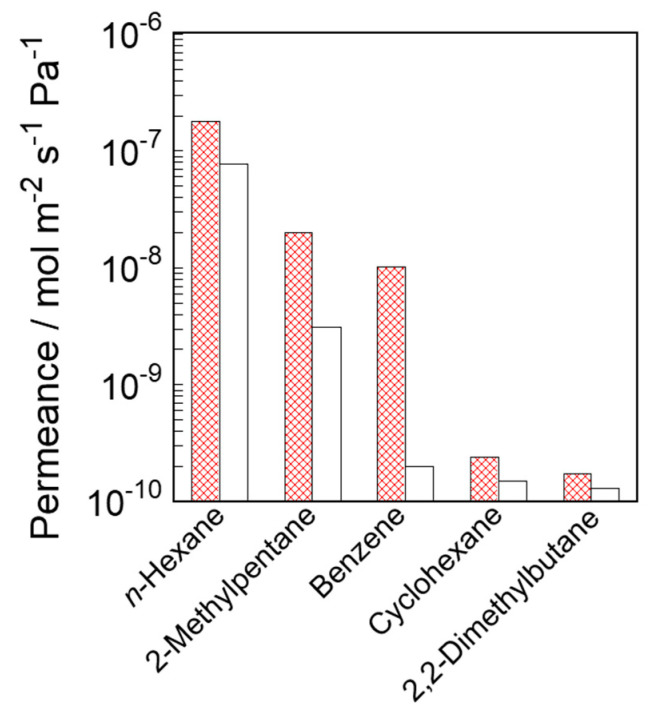
Hydrocarbon permeances through the silicalite-1 membrane in unary systems. Shaded and open bars correspond to S-1_S_ and S-1_M_, respectively.

The permeances of C_6_ hydrocarbons with various molecular sizes were studied at 573 K to evaluate the permeation properties of the prepared silicalite-1 membranes. *n*-Hexane, 2-methylpentane, benzene, cyclohexane and 2,2-dimethylbutane were used as feed substances. Figure 7 shows the results of the permeation tests in these unary systems.

The order of hydrocarbon permeances through both silicalite-1 membranes corresponded to the order of the molecular sizes. The *n*-hexane permeances were the largest among the C_6_ hydrocarbons due to their slim shape. The *n*-hexane permeances through S-1_S_ and S-1_M_ were 1.78 × 10^−7^ and 7.67 × 10^−8^ mol m^−2^ s^−1^ Pa^−1^, respectively. The 2-methylpentane permeances through S-1_S_ and S-1_M_ were the second largest, at 1.70 × 10^−8^ and 3.13 × 10^−9^ mol m^−2^ s^−1^ Pa^−1^, respectively. In contrast, the cyclohexane and 2,2-dimethylbutane permeances were extremely low; they hardly entered the original micropore of the MFI-type zeolite, owing to their bulky shapes. Since both membranes had few defects, these large, bulky molecules were not able to penetrate the membranes.

As a result, both silicalite-1 membranes exhibited excellent separation performances from the viewpoint of the molecular sieving effect. The ideal selectivities for *n*-hexane and 2,2-dimethylbutane through S-1_S_ and S-1_M_ were 1030 and 590, respectively. An interesting feature was that the benzene permeances were significantly different between these membranes. The benzene permeance through S-1_S_ was 1.01 × 10^−8^ mol m^−2^ s^−1^ Pa^−1^, which was ca. 50 times larger than that through S-1_M_.

## 4. Discussion

### 4.1. Effect of Synthesis Conditions on Crystal Structures and the Appearance of Membranes

In this section, we will discuss the effect of synthesis conditions on crystal structures and the appearance of membranes, S-1_S_ and S-1_M_, from the results of XRD and microscopic observation, as shown in Figure 2, Figure 3 and Figure 4.

While the intensities of the diffraction pattern of S-1_M_ were tripled for ease of viewing, the XRD patterns of the two kinds of membranes were identical to that for the typical silicalite-1 powder, indicating that the obtained membranes were purely composed of the MFI-type zeolite. Because these XRD measurements provide information from the vicinity of the support surface, the intensities of the diffraction peaks are considered to be greatly influenced by the membrane thickness. Therefore, we hardly discuss the differences in the crystallinity between these two membranes from the intensities; the degree of crystallinity should be discussed based on the micropore volume evaluated by N_2_ adsorption, which will be described in the following section.

Table 1 lists the particle sizes of the seed crystals, the weight of the seed supported and the number of supported seeds. The number of supported seed was calculated as follows:

Number of supported seed = *W_S_* · *V_S_*^−1^ · *ρ*^−1^ (3)

where *W_S_* is the weight of the seed supported (g), *V_S_* is the volume of a seed crystal (cm^3^) and *ρ* is the density of the silicalite-1 crystal (g cm^−3^). The volumes of both seed crystals were calculated assuming 270 and 100 nm spheres. In the case of seeding through dip-coating with small crystals, a large amount of seed was loaded judging from the weight gain, although only a few crystals were observed on the outer surface. This result suggested that most of the seed crystals were embedded in the voids of the porous support because they were smaller than the support pores, possibly because the slurry would be strongly pulled into the support pores during the dip-coating step by the capillary force.

Here, we focus on the difference in the crystal sizes in the membranes. The difference is probably due to the difference in the seeded and crystallization conditions. The number of seed crystals per unit of surface area in the seeded support for S-1_M_, 1.6 × 10^15^ m^−2^, was much larger than that for S-1_S_, 1.1 × 10^14^ m^−2^. In S-1_S_, the small number of nuclei could lead to the formation of large crystals, because Si contained in a mother solution was shared during the course of the secondary growth process by each nucleus in a batch-wise reactor. In addition, from the SEM images shown in Figure 3 and Figure 4, we estimated the number of seed crystals and grown crystals in S-1_S_. Because the number of seed crystals was almost the same as that of the grown crystals, we concluded that it would be rather difficult for heterogeneous nucleation to occur during the course of S-1_S_ formation. As a result, we considered that there were fewer grain boundaries across the direction of permeation in S-1_S,_ but a larger number of grain boundaries that disturbed molecular permeation in S-1_M_.

### 4.2. Micropore Volume, Effective Pore Size and the Permeation Property

In this section, we will describe permeability and pore-connectivity for these two silicalite-1 membranes. We will discuss the differences in micro-structures among two kinds of membranes and powders from the results of nano-permporometry, adsorption measurement and permeation tests.

We evaluated the amounts of non-zeolitic pathways and the through-pore size in the membranes from the results of nano-permporometry. The size of the pores plugged by *n*-hexane condensation at *p* · *p_S_*^−1^ = 8.0 × 10^−3^ was calculated to be 0.56 nm using the Kelvin equation, the pore size of which is almost the same as the original pore size of the MFI-type zeolite. Therefore, the helium permeance above 8.0 × 10^−3^ of *p* · *p_S_*^−1^ corresponds to the amount of non-zeolitic pathways that are larger than the zeolite pore. For both silicalite-1 membranes, helium hardly penetrated above *p* · *p_S_*^−1^ = 1.0 × 10^−2^, and these permeances were below the limit of quantification. These results clearly showed that both silicalite-1 membranes were compact and had few non-zeolitic pathways.

At lower relative pressures, helium permeances through these two types of membranes were very different. In S-1_M_, *n*-hexane blocked the helium permeation at a lower relative pressure, 3 × 10^−3^ of *p* · *p_S_*^−1^, compared with S-1_S_. Generally, because of the enhanced interaction between adsorbate and adsorbent, adsorption and condensation tend to occur in smaller pores at lower relative pressure. From the results of nano-permporometry for S-1_M_, we can conclude that S-1_M_ had a narrower part where *n*-hexane could easily condense. In contrast, helium permeated up to 8.0 × 10^−3^ of *p* · *p_S_*^−1^ through S-1_S_, indicating that the through-pore size of S-1_S_ was close to the original pore size of the MFI-type zeolite.

Here, we explain the reason for the difference in the adsorbed amounts among the silicalite-1 powder and membranes, based on the results shown in Figure 6. If all micropores are accessible, the amounts adsorbed on the silicalite-1 powder and these membranes should be the same. However, the amounts adsorbed on the membranes were smaller than those of the silicalite-1 powder, as shown in Table 2. The mismatch of the micropore may occur at the crystal interfaces formed through the collision of growing crystals during the course of membrane formation, as schematically drawn in Figure 1, where the micropores can be narrowed and/or obstructed, thus reducing the accessible volume.

In addition, the difference in the adsorbed amounts of 2-methylpentane among the three kinds of adsorbates was especially large, indicating that the larger the molecule, the more strongly it tended to be affected by the narrowness of pores. Since the adsorption measurements evaluated not only the through-pores but also the obstructed pores, the results of the adsorption measurement did not accurately indicate the narrowing of the through-pores, although they agreed well with the results of the nano-permporometry.

We defined pore-connectivity based on the results of the adsorption tests. The molecular sizes and the relative adsorbed amounts were plotted in Figure S1 in the Supplementary Materials. The fitting curves in Figure S1 that were extrapolated up to 0.55 nm, the size of the micropore of the MFI and the *y* values at 0.55 nm were defined as pore-connectivity. The pore-connectivity is the percentage of the micropores that are not narrowed and/or blocked but maintain their original pore size. Based on this definition, we evaluated the pore-connectivity of S-1_S_ and S-1_M_ to be 60 and 47%, respectively.

We consider the significance of the differences shown in Figure 7 for the permeabilities through the two silicalite-1 membranes. Compared to S-1_M_, S-1_S_ exhibited a higher permeation performance for *n*-hexane, 2-methylpentane and benzene, suggesting that S-1_S_ had relatively smaller permeation resistance. The permeances of the hydrocarbons through S-1_S_ were larger than those through S-1_M_, although the membrane thickness of S-1_S_ defined by the FE-SEM and the membrane weight was thicker than those of S-1_M_. For example, n-hexane and 2-methylpentane permeances through S-1_S_ were 2.3 and 6.4 times larger than those through S-1_M_, respectively. Moreover, the differences in permeance tended to be larger with increasing molecular size. Since the size of benzene is very close to the pore size of MFI, the fact that benzene hardly permeated through S-1_M_, suggests that the effective through-pore size of S-1_M_ is narrower than the original pore size. This difference in benzene permeance between S-1_S_ and S-1_M_ is in good agreement with the results of the nano-permporometry and the adsorption test.

Richter et al. reported the effect of the addition of alcohols in the synthesis solution [36]. In this paper, they have reported the formation of an additional non-zeolitic pore system in a zeolitic layer through the addition of alcohol, leading to the improvement of the permeability of the ZSM-5 membrane. When the water in the synthesis solution was substituted by the same molar amount of short-chain alcohol, the permeances of 1-butene increased remarkably but the 1-butene/*i*-butene selectivity decreased only slightly. As in their study, we observed larger permeance through S-1_S_, which was prepared by the addition of ethanol; the presence of ethanol in the synthesis solution may contribute to the improvement of the permeation performance.

**Figure 8:**
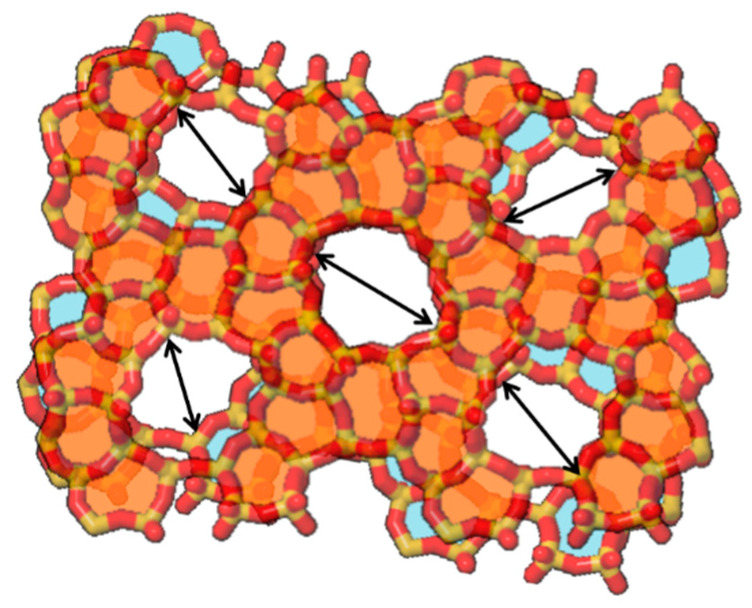
Stacking of two MFI-type zeolite structure sheets viewed from the *b*-axis with 5-degree rotation.

Thus, we suppose that the narrowing and obstruction of the zeolite pores were caused by the mismatch of micropores at the interfaces of the crystals along the grain boundaries. As mentioned above, grain boundaries are formed when crystals collide with each other during the course of the secondary growth process. Figure 8 illustrates two stacked sheets of MFI-type zeolite structures rotated by 5 degrees and viewed from the *b*-axis direction. One can clearly observe that the pore-connectivity becomes worse through only 5 degrees of twisting. Whereas we do not have concrete evidence about such mismatch of micropores at the crystal interface along the grain boundary, pore-narrowing and -obstruction were strongly suggested to occur through the results of the characterizations described above.

In S-1_M_, due to a larger number of crystals, pore-obstruction and -narrowness occur with high frequency at these grain boundaries. As a result, the through-pore size and pore volume determined by the nano-permporometry and the adsorption measurement were smaller than those of the typical silicalite-1 powder. On the other hand, since S-1_S_ had less crystal stacking and fewer grain boundaries across the direction of the molecular permeation, it seems that the through-pore size and pore volume of S-1_S_ were relatively close to those of the silicalite-1 powder. Accordingly, in order to reduce permeation resistance, eliminating grain boundaries and improving pore-connectivity is an effective strategy of membrane preparation.

## 5. Conclusions

Pore-connectivity in two types of silicalite-1 membranes was investigated through using nano-permporometry, adsorption tests and vapor permeation tests. These results showed that a membrane with many grain boundaries in the direction across the molecular permeation resulted in lower pore-connectivity, smaller effective pore size and lower permeation performance. In contrast, a membrane with fewer grain boundaries exhibited relatively better pore-connectivity and permeation performance, indicating that the narrowness and obstruction of micropores occurred at the grain boundaries of the silicalite-1 crystals. In particular, there was a 50-fold difference in benzene permeance between the two types of membranes, with the pore-connectivity of 60% and 47%. In summary, it is important to reduce grain boundaries and improve pore-connectivity to develop an effective preparation method of obtaining a highly permeable membrane.

## Figures and Tables

**Table 1:** Seeding and membrane features of silicalite-1 membranes.

	Particle Size /nm	Seeded Weight /g m^−2^	Number of Seeds /10^14^ m^−2^	Membrane Weight /g m^−2^	Membrane Thickness /mm
S-1_S_	270	2.8	1.1	68.6	4.0
S-1_M_	104	1.5	16	43.7	2.5

**Table 2:** Amounts adsorbed in the micropores of silicalite-1 powder and membranes.

	Adsorbed Amount in Micropore /cm^3^ (STP) g^−1^			Relative Adsorbed Amount /%			Pore-Connectivity /%
	N_2_	*n*-Hex	2-MP	N_2_	*n*-Hex	2-MP	
Powder	92.6	33.0	19.9	100	100	100	100
S-1_S_	82.1	21.1	12.0	88.7	63.9	60.3	60
S-1_M_	58.4	16.5	9.52	63.1	50.1	47.8	47

## Supplementary Materials

The following are available online at https://www.mdpi.com/article/10.3390/membranes11060382/s1, Figure S1: Relationship between molecular sizes and relative adsorbed amounts of *n*-hexane and 2-methylpentane on S-1_S_ and S-1_M_.

## Nomenclature

*A*	Effective membrane area [m^2^]
*D_K_*	Kelvin diameter [m]
*ν*	molar volume [m^3^ mol^−1^]
*p*	Partial pressure [Pa]
*p_S_*	Saturated vapor pressure [Pa]
*θ*	Contact angle [degree]
*R*	Gas constant [J mol^−1^ K^−1^]
*σ*	Surface tension [N m^−1^]	
*T*	Temperature [K]
*u*	Flow rate [mol s^−1^]
