# Supplementary material for: Contribution of Pore-Connectivity to Permeation Performance of Silicalite-1 Membrane; Part I, Pore Volume and Effective Pore Size
Source: Membranes (Basel). 2021 May 24;11(6):382. doi: 10.3390/membranes11060382 (PMC8225034; doi:10.3390/membranes11060382)
Supplement: Supplementary file 1 [file membranes-11-00382-s001.zip › membranes-1224424-supplementary.pdf]

Supplementary Materials

Title: Contribution of Pore-Connectivity to Permeation Performance of Silicalite-1 Membrane; Part I, Pore Volume and Effective Pore Size

Authors: Motomu Sakai[1]*, Yukichi Sasaki[2], Takuya Kaneko[3], and Masahiko Matsukata[1,2,4]

Affiliation:
1 Research Organization for Nano & Life Innovation, Waseda University, 513 Waseda-Tsurumaki-cho, Shinjuku-ku, Tokyo 162-0041, Japan
saka.moto@aoni.waseda.jp
2 Nanostructures Research Laboratory, Japan Fine Ceramics Center, 2-4-1 Atsuta-ku, Nagoya-shi, Aichi 456-8587, Japan
3 Department of Applied Chemistry, Waseda University, 513 Waseda-Tsurumaki-cho, Shinjuku-ku, Tokyo 162-0041, Japan
4 Advanced Research Institute for Science and Engineering, Waseda University, 513 Waseda-Tsurumaki-cho, Shinjuku-ku, Tokyo 162-0041, Japan

***Corresponding author:**
Phone number: +81-3-5286-3850
e-mail address: saka.moto@aoni.waseda.jp
Postal address: 513 Waseda-Tsurumaki-cho, Shinjuku-ku, Tokyo 162-0041, Japan

Fig. S1 shows the relationship between molecular sizes and relative adsorbed amounts on S-1$_S$ and S-1$_M$. We defined a pore-connectivity as follows. The fitting curves in Fig. S1 extrapolated up to 0.55 nm, a size of micropore of MFI, and the relative adsorbed amount at 0.55 nm were defined as a pore-connectivity. The pore-connectivity means that a ratio of the micropore remain the original pore size without pore-narrowing and -obstruction. The pore-connectivities of S-1S and S-1M were 60 and 47%, as shown in Fig. S1.

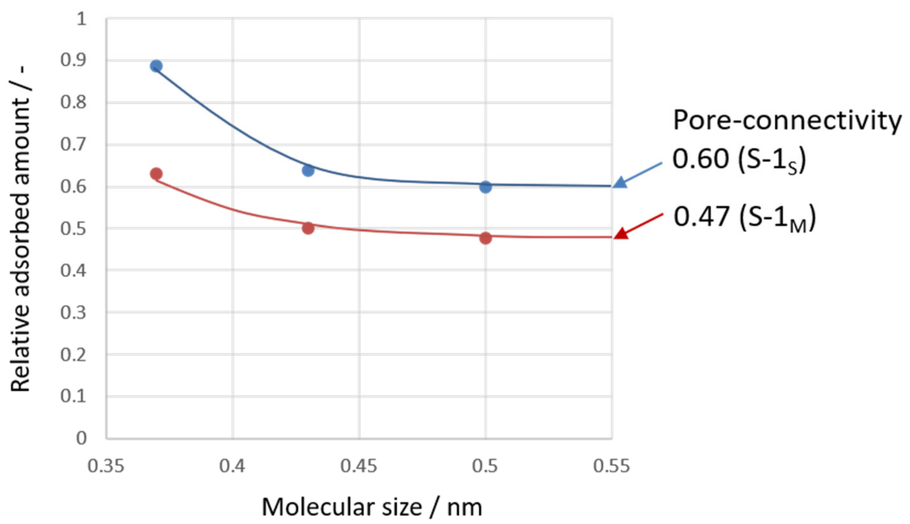

Figure S1 Relationship between molecular sizes and relative adsorbed amounts of *n*-hexane and 2-methylpentane on S-1_S and S-1_M.
